# Discussion on Diphtheria

**Published:** 1866-07

**Authors:** 


### Proceedings of Societies.

### PROCEEDINGS OF THE SEVERAL SECTIONS OF THE AMERICAN MEDICAL ASSOCIATION, DURING THE RECENT MEETING IN BALTIMORE.

The greater part of the following report of the doings in the several Sections is copied from the *New York Medical Record, for May, 1866*:—

### SECTION ON PRACTICAL MEDICINE AND OBSTETRICS.

### DISCUSSION ON DIPHTHERIA.

The section was organized by the appointment of Dr. Lake J. Tefft, of New York, Chairman, and Dr. W. B. Bibbins, of New York, Secretary.

Dr. H. D. Holton, of Putney, Vermont, Chairman of the Committee on Diphtheria, as it had prevailed in the United States, made a report by reading an elaborate and interesting paper. He gave a history of the disease from its first appearance in this country, more than a century since. He insisted that diphtheria should not be confounded with follicular tonsilitis and kindred affections; in fact nothing should be dignified with the name that did not present the characteristic exudation, with swelling of the cervical glands, etc. Neither should it be confounded with scarlatina or croup. Tables were presented showing the fatal months, the proportion of deaths at various ages of the three diseases. The disease was divided into diphtheria simplex, diphtheria maligna or gangrenosa, and tracheal diphtheria. He entered at some length into the discussion of the sequelæ, stating that they were paralysis, rheumatism, and general cachexia.

After discussing the various plans of treatment, he divided it into local and general. Such mild cathartics should be used as the case seemed to require, although nothing like active purgation should be indulged in. Chlorate potassa should be given pulverized with an equal amount of sugar, a little placed in the mouth, and allowed to run down the throat, that the local as well as the constitutional effect might be obtained.

Sulph. quinin. and tr. ferri chloridi should be used as occasion required; also alcoholic liquors, either alone or combined with cinchona bark; wine whey, milk punch, and beef-tea were to be used to support the patient when a nourishing diet could not be taken.

The local applications should be such as would prevent the absorption of fluid portions of the exudation. For this purpose persulphatum ferri was recommended; also the following: ℞. Tr. ferri chlorodi, chlorat. potassæ *aa* ℥ j; glycerine pure, ℥ ij. Misce. Either of these to be carefully applied with a soft brush. Externally, an infusion of hops in lye of wood ashes would be found the best for any hot fomentation.

The following deductions were made: 1st. That diphtheria has occurred as an epidemic from time to time from the first settlement of this country; 2d. That it is a distinct disease, on no account to be confounded with scarlatina or croup; 3d. That it is particularly a disease of childhood, although it exempts no age; 4th. That it is communicable to that degree that it is the duty of every physician to separate the infected from those that are well, particularly in children; 5th. That it is a disease primarily affecting the blood, consequently the treatment to be effective must be not local but general, and of such a nature as to eradicate or neutralize the poison; such local treatment should, however, be used as will prevent the absorption of fluid portions of the exudation; 6th. That when the exudation has invaded the trachea, the only hope of saving the patient is in tracheotomy.

On motion of Dr. Ellsworth Eliot, of N.Y., the report was referred to the Committee on Publication.

Dr. King wished to take exception to the remark made in this paper that when the membrane is thrown off from the trachea the patient will almost always get well. He had seen two cases, one a man and the other a child, in which the membrane had been thrown off and both had died. He could not believe that the cast-off membranes were ever re-absorbed. He stated that he had ceased to use local applications for anything more than their antiseptic effects; and for this purpose he thought that the solution of common salt was as good as anything else.

Dr. Gallagher, of Pittsburg, Pa., expressed himself much pleased with the report. He regretted, however, that the author had made no reference to the use of alum as a local application. He had used that remedy with the happiest effects, and had more confidence in it than all the others. He had used also with great satisfaction the ordinary "hard cider," with a view of assisting the elimination of the morbid materials by the kidneys.

Dr. Worthington Hooker, of New Haven, stated that there was a great tendency in the profession to run into extremes in reference to treatment. He was not disposed to think that any disease could be successfully treated by following any one plan to the exclusion of others. The really rational plan was to follow all the indications in the particular case as they might arise. There were many marked distinctions between croup and diphtheria, but he chose only to refer to the want of that severe constitutional disturbance in the former which is never absent in the latter disease.

Dr. McCollum, of Vermont, stated he had seen 200 cases of the disease in his county within the last five years. He had seen three or four cases recover after the membrane had been cast off. He never used gargles except as disinfectants. As regards ice, he had noticed benefit from its use in a great many cases. He had also been in the habit of allowing his patients to inhale the vapor of acetic acid, and with marked benefit.

Dr. Gross, of Pittsburg, stated that he had had a large experience in the treatment of diphtheria in his district, and had come to the conclusion that the best means of treatment consisted, first in the use of calomel as an antiphlogistic, followed by the very free use of chlorate of potash. He had seen 400 or 500 cases of the disease since he instituted this plan of treatment, but had not met with a single fatal case. He also, in conjunction with the foregoing remedies, made use of the solution of nitrate of silver (20 grains to the ounce) as a local application, as well as saturated solutions of alum and lemon-juice on crushed ice.

Dr. Bibbins, of N.Y., stated that some time since the question was asked him by Prof. A. Clark—What was the number of cases of diphtheria which he had met with in dispensary practice? Dr. Bibbins, before he was prepared to answer such a question, wished to know from Dr. Clark what he understood to be true diphtheria—whether the essential symptoms of the disease were considered to be adenitis, false membrane, fever, and depression of the vital powers. "Of course," replied Dr. Clark, Dr. A. Jacobi, who had studied this subject very thoroughly, has published the fact that these symptoms are always essential to the disease. A certain proportion of cases must be distributed to each individual; and such being the fact, they could not but believe that when gentlemen report such a large number of cases as occurring in their practice they do not take into account the necessary symptoms of diphtheria; that a great many report cases which they call diphtheria, which other practitioners are not willing to admit as such. He remarked that the average mortality of true cases of diphtheria among dispensary patients was thirty-three per cent., and did not suppose that under any treatment this mortality could be much decreased. Dr. A. Clark had reported the same rate of mortality. Dr. Bibbins was led to believe, from the cases which he had met with, that the tonic and supporting plan of treatment was the only reliable one.

Dr. Shumway, of N.Y., had used with great benefit, as local applications, the nitrate of silver stick, the chlorate of potash, and the chlorate of ammonia. He apprehended that the great difficulty with many patients that were convalescent was not that they did not get medicine enough, but that there was paralysis of the throat which prevented them from swallowing them. In those cases he had used nutritive injections into the stomach with very good results.

Dr. Davis, of Ill., spoke in this connexion of blood-poisoning in many diseases, diphtheria among the rest. He thought that there was yet a great deal to be learned with reference to the true pathology of these so-called blood diseases. The query had arisen with him whether diphtheria really depended primarily upon a poison in the blood, or whether the altered condition of the blood itself, instead of being primary, was itself a secondary consequence of a prior change in the properties of the organized tissues of the human body. He discussed this question at a considerable length, showing the probability that there might be such a fundamental error in nutrition, as that assimilation or disassimilation was so interfered with that the blood was poisoned in consequence.

Dr. Hewitt remarked that he had great faith in the solvent power of both alum and chlorate of potash on the diphtheric membrane. He had taken pieces of the membrane that had been separated, and had dissolved them entirely in solutions of these salts. His treatment was to take a glass tube, pass it back into the fauces, and inject one or other of the solutions directly against the affected parts.

Dr. McIntyre, of Concord, N.H., was of the opinion that if there was any error in nutrition, it was secondary to the formation of the membrane in the throat, which by obstruction to the respiration interfered with the proper oxidation of the blood. This he considered to be the starting-point of the constitutional trouble. For the purpose of aiding the system in the elimination of the poison in the later stages of the disease he had used colchicum seed, guaiac, and nitrate of potash, with much satisfaction.

Dr. Taylor, of Iowa, did not think that there was any good to be attained by suggesting this and that remedy for a disease which had existed in every section of the country; for different remedies must be used in different localities, and he thought the best guide for any general plan of treatment was to be found in a proper, thorough, and enlightened view of the pathology of the disease.

The meeting then adjourned.

SECOND SESSION.

Dr. Lake J. Tefft in the chair.

The second meeting of the Section was held at the Dental College, corner of Hanover and Lombard streets, on Wednesday, May 2, 1866, at 3.45 P.M.

The minutes of the last meeting were read and approved.

Dr. James J. Levick, of Philadelphia, read the "Report of the Special Committee on Spotted Fever."

On motion of Dr. Henry D. Holton, of Putney, Vt., the report was referred to the Committee on Publication, with a recommendation for its publication in the Transactions of the Association.

